# A qualitative study of the factors impacting implementation of the national action plan to contain antimicrobial resistance (2016–2020) in medical institutions

**DOI:** 10.1186/s12913-023-10404-y

**Published:** 2024-01-22

**Authors:** Yun Tao, Ying Wang, Yu Zhang, Yong Han, Jiajia Feng, Hong Cheng, Likai Lin

**Affiliations:** 1 Hospital Management Institute of Wuhan University, Zhongnan Hospital of Wuhan University, Wuhan, 430071 Hubei China; 2 Department of Infectious Diseases, Zhongnan Hospital of Wuhan University, Wuhan, 430071 Hubei China; 3 Department of Pharmacy, Zhongnan Hospital of Wuhan University, Wuhan, 430071 Hubei Province China

**Keywords:** Antibacterial agents, Antimicrobial resistance, The national action plan, Medical institution

## Abstract

**Objective:**

Antimicrobial resistance (AMR) has emerged as a serious global public health crisis. In response, 2016, 14 ministries in China, under the leadership of the National Health Commission, collaboratively issued the National Action Plan (NAP) to Contain Antibacterial Resistance (2016–2020). The NAP outlines strategies for medical institutions to adopt stewardship and implement AMR control. The purpose of this study was to comprehend stakeholders’ perceptions of the NAP and explore the factors that influence its implementation in medical institutions.

**Methods:**

Semi-structured interviews were conducted with practitioners from medical institution in March and April 2021. Interviews were audio-recorded, transcribed and analyzed using thematic analysis via the framework approach.

**Results:**

Twenty practitioners, representing diverse roles (4 administrators, 7 clinicians, 3 microbiologists, 3 pharmacists, 3 nosocomial infection management personnel) from seven institutions, participated in the study. Substantial efforts have been undertaken to regulate the rational use of antibiotics and enhance the management of hospital infections. Participants demonstrated awareness and concern regarding antimicrobial resistance, with widespread support expressed for the NAP. Among all professions, there were varying opinions on whether they felt restricted in their daily work. The tertiary hospitals have established multidisciplinary cooperation mechanisms. Six main themes were identified as both barriers and facilitators to the implementation of the NAP in the medical institutions: individual factors, leadership, multidisciplinary collaboration, patient factors, training and culture. The capacity for administrative attention is constrained or limited, poor enforcement of guidelines, insufficient specialist staff and the liability pressure on clinicians were perceived barriers. To containing AMR in medical institutions, management of hospital infections, the public’s knowledge of antibiotics’ usage, routine education and multidisciplinary support would be facilitators.

**Conclusions:**

Practitioners from medical institutions were highly supportive for the NAP. Consideration of practitioners’ perceived barriers and facilitators might enhance implementation of the NAP to contain antimicrobial resistance.

**Supplementary Information:**

The online version contains supplementary material available at 10.1186/s12913-023-10404-y.

## Background

Antimicrobial resistance (AMR) has caused socioeconomic burden and becoming public health crisis. The World Bank report forecasts that if AMR is not well controlled globally, healthcare costs may increases up to $1 trillion per year by 2050 [1]. During the G20 Summit in 2016, agreement has been made on the importance of controlling AMR. In 2015, a global action plan on AMR control was released by the WHO, and requires all member countries to develop their own action plan to contain AMR. The Chinese government have been actively involved in tackling AMR, the National Health Commission led the issue of the National Action Plan (NAP) to Contain Antibacterial Resistance (2016–2020) [2]. The NAP emphasizes the heavy responsibility of medical institutions and health authorities in the management of antibiotics and contain AMR. The government has integrated antimicrobial control into hospital management systems and promoted the implementation of actions in medical institutions.

Here, the NAP comprises strategies in health administration and medical institutions for regulate the rational use of antibiotics in medical practice in the hospital management system. The overarching aim was to contribute to the advancement of China’s healthcare objectives, ultimately fostering a healthier nation. In pursuit of this objective, a selection of five provinces including Hubei was made for inclusion in the evaluation program in 2021 urged by National Health Commission. Hubei Province has made great efforts to implement the national action plan. Hubei Provincial Health and Family Planning Commission have issued measures for the Graded Administration of Clinical Use of Antibiotics in Medical Institutions of Hubei Province in 2012. This study was designed to understand the measures taken by the health administration and medical institutions for NAP, to know information on the use of antimicrobial drugs of AMR in Hubei, understand practitioners’ perceptions on the NAP, to investigate factors influencing the NAP implementation in medical institutions.

## Methods

### Setting and research design

Hubei Province, situated in central China, occupies a strategic location. The annual average income per capita in Hubei positions it within the middle tier among all provinces. In the year 2022, Hubei Province achieved a total gross domestic product (GDP) of 7,374 billion US dollars, with a corresponding GDP per capita of 12,648 US dollars—comparatively higher than the national average of 11,769 US dollars. Notably, approximately 5.89% of Hubei’s gross domestic product was allocated to the healthcare sector.

This was a qualitative, multicenter study performed in seven healthcare facilities which including 4 tertiary hospitals, 1 secondary hospital and 2 community healthcare centers.

### Study participants

The interviewee including subjects in relation to practicing and implementation of the NAP: clinicians, pharmacists, microbiologists, infection control practitioners, hospital administrator and official of health administration. An interview guide was developed based on literature review and advice from specialists, we explored the experience and perspectives of the NAP implementation in medical system, the facilitators and barriers influencing the NAP implementation from the interviewee (see supplementary material). Purposive (through professional networks) and snowball sampling were utilized to recruit participants [3, 4].

### Interview

Experienced personnel conducted the semi-structured and structured interviews in March and April 2021. The interview questions faced by respondents in the same field are standardized. Each interview lasts about 20–30 min. The interviews were recorded by the researchers and transcribed verbatim. Transcripts were verified by interviewers.

### Data source

One of interviewee have provided the utilization rate of antibiotics prescribed by medical institution which is collected from Hubei Province antibiotics clinical application monitoring network. There are 81 member units officially reporting data to the monitoring network in Hubei Province, of which 56 are tertiary hospitals and 25 are secondary hospitals. The interviewee agreed to include the data in the article.

At the general level, the use of antibiotics was investigated. The proportion of antibacterial drug use in outpatients is equal to the number of patients who use antibacterial drugs divided by the total number of patients in the same period. The utilization rate of antibiotics in hospitalized patients is equal to the total number of discharged patients using antibiotics divided by the total number of discharged patients in the same period. No matter how many antibiotics (including different dosage forms) were used in a case, only one case of antibiotic use was counted. The data are for the whole year.

### Analysis

A framework approach is adopted for thematic analysis. Themes were identified and categorized. The interviews were coded independently by two researchers. One reviewer used NVivo 12 qualitative data analysis software, and the other coded it manually. Through discussion among reviewers, a consensus on topic was established. The researchers obtained official permission from the participating hospitals, and all participants signed informed consent prior to participating in the study.

## Results

### Implantation experience in Hubei province in central China

#### Strategies to combat AMR in the Hubei health system

##### Regulatory measures

In 2018, the Hubei local health administration department introduced regulations concerning the clinical use of antibiotics. They also issued a notification in the same year to enhance the reporting and monitoring of bacterial drug resistance in Hubei, aimed at promoting rational drug usage.

##### Graded management of clinical antimicrobial agents

The Hubei Province Health Administration Department has released a “Catalogue of Graded Management of Clinical Antimicrobial Agents” for the circulation of antimicrobial agents. According to their management approach, healthcare institutions at all levels are required to implement a classification system for managing antimicrobial agents.

##### Special antibiotic use rectification campaign (2015–2017)

This campaign focused on ensuring responsible management of rational antimicrobial agent application. It aimed to improve the overall clinical application of antimicrobial agents, including factors such as utilization rates and intensity among inpatients, prophylactic antibiotic use in specific surgical procedures and interventional treatments, special antibiotic usage, and the prescription ratio of antimicrobial agents in outpatient and emergency departments.

##### Clinical pharmacy quality control center and expert committee

To promote rational use of antimicrobial agents in medical institutions, the Hubei health administration department established the Hubei Expert Committee of Drug Rational Use announced in 2017 in Wuhan City. This Committee provide professional support for drug policies in the province, formulates technical guidelines, standards and assessment systems for rational drug use in clinical practice, and conducts specific guidance, educational, training, and promotional activities.

The Clinical Pharmacy Quality Control Center’s main responsibility is to coordinate experts for guiding, supervising, inspecting, and evaluating the quality control of antibiotic usage in medical institutions and providing suggestions for improvement.

##### Surveillance networks for antibiotics use and bacterial resistance

Recently, the number of member hospitals in Hubei’s surveillance networks has grown to over 50, including secondary and tertiary hospitals. Typically, the pharmaceutical department reports antibiotic usage, while the microbiology laboratory reports antibiotic resistance.

##### Public education

Efforts for health education are conducted through various social media and traditional media channels. Additionally, there is an annual event known as “Hubei Provincial Rational Drug Use Publicity Week.“

**Fig. 1:**
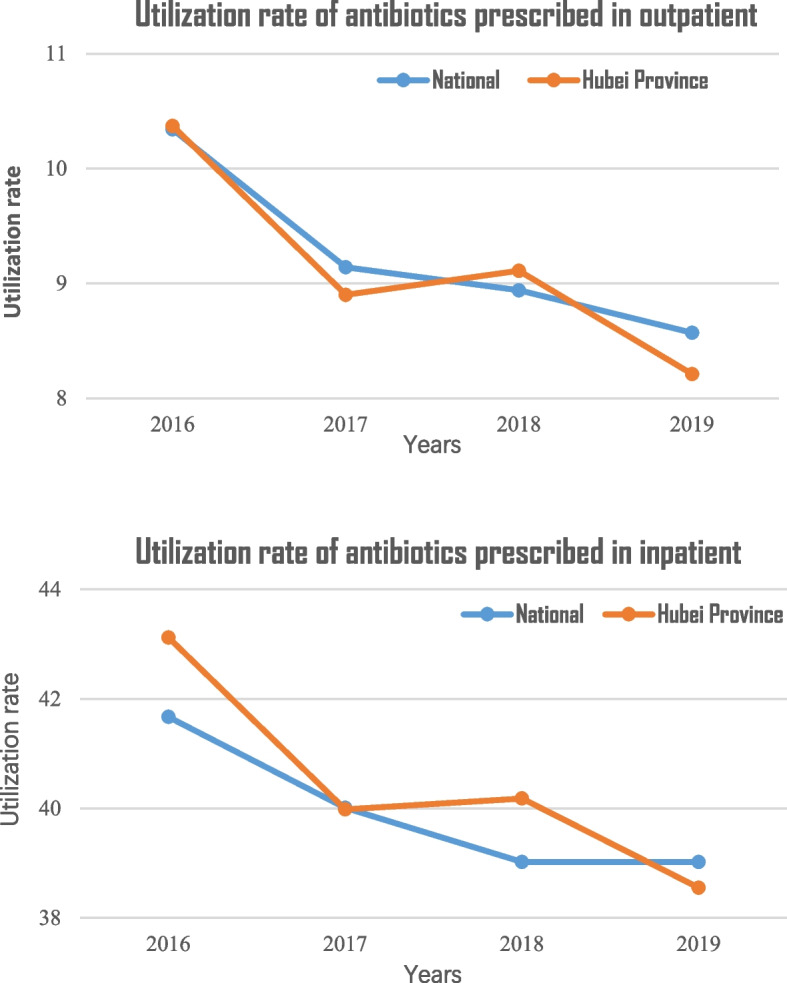
Utilization rate of antibiotics prescribed in medical institution in Hubei Province

##### Antibiotic prescriptions in medical institutions

From 2016 to 2019, both nationally and in Hubei, the prescription rate of antibiotics for outpatients and inpatients has decreased. China has seen a declining trend in antibiotic prescription rates over the past few decades, with the rate now significantly lower than before (see Fig. 1).

Based on a systematic review of antibiotic use studies in China, outpatient antibiotic prescriptions accounted for 50.3% from 2000 to 2012 [5]. Policy interventions have played a significant role in reducing antibiotic use, as demonstrated by a retrospective study in a tertiary hospital in Beijing. Between 2011 and 2016, the proportion of antibiotics prescribed in outpatient and inpatient departments decreased (from 19.38 to 13.21% and from 64.34 to 34.65%, respectively), with the ‘National Stewardship in the Clinical Utilization of Antimicrobial agents (2011)’ having a key impact on reducing antibiotic use [6].

#### Actions within medical institutions

The efforts of medical institutions to contain antimicrobial resistance mainly revolve around two parts: antimicrobials stewardship and infection prevention. One of the major strategies in the NAP is to strengthen the construction of AMR control system especially in strengthening the management of hospital infections. The emergence of drug-resistant organisms in hospitals is a direct consequence of healthcare interventions, so the implementation of strong infection prevention and control guidelines is critical. There is a synergy between infection prevention and antimicrobial stewardship. Targeted coordination and prevention strategies are critical to stopping the spread of multi-drug-resistant organisms. The tertiary hospitals from the investigated hospitals have established committee on the Rational Use of Antimicrobial Drugs and Hospital Infection Management Committee, hospital leaders serve as committee directors.

**Fig. 2:**
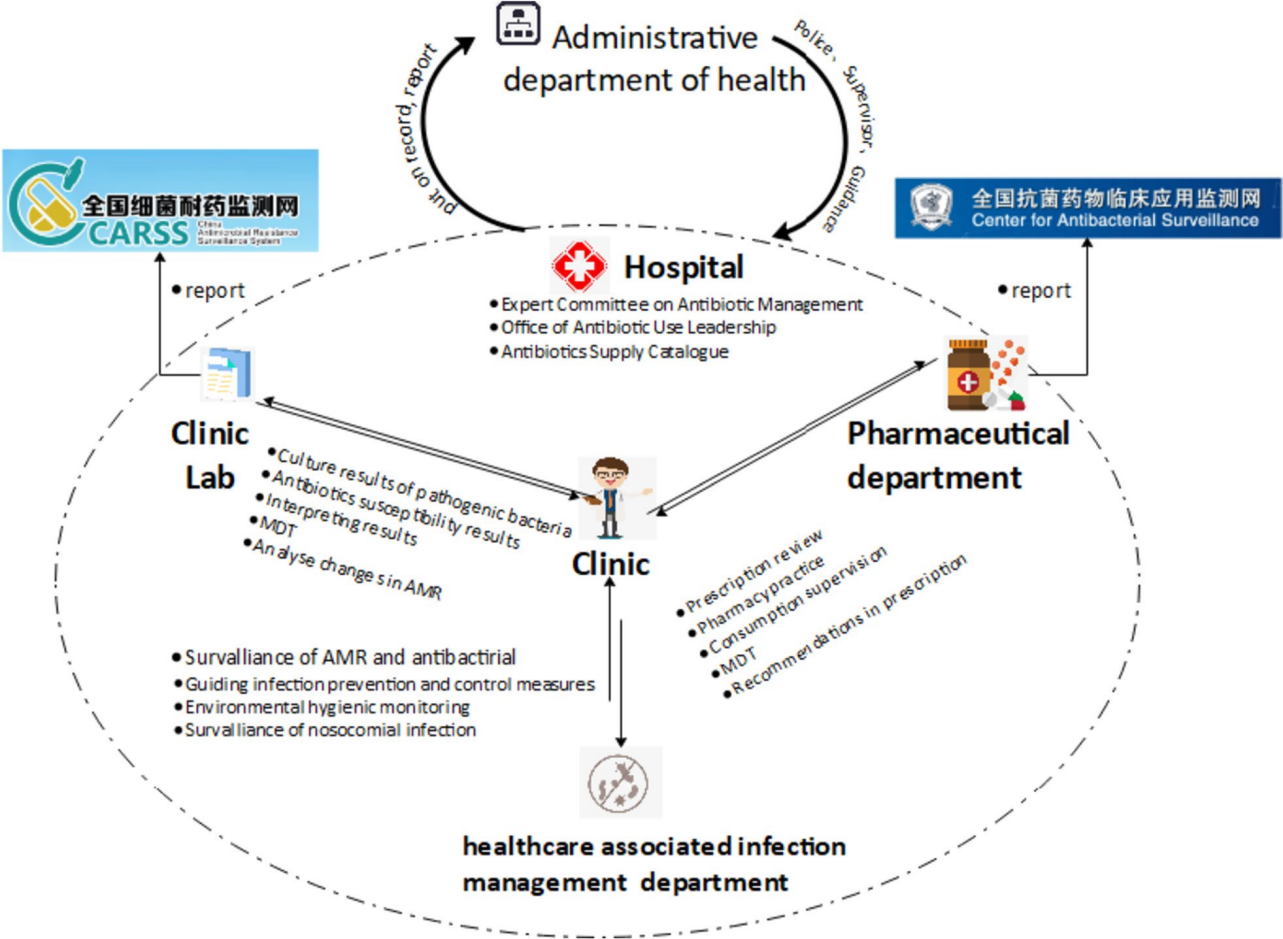
Multidisciplinary cooperation mechanisms in hospitals

A multidisciplinary cooperation mechanism has been established in the tertiary hospitals of the surveyed hospitals. The multidisciplinary working group consists of clinical departments, clinical microbiology, pharmacy and hospital infection management (Fig. 2).

Antibiotics are classified into special use grade, restricted use grade and unrestricted use grade in medical institutions. Special purpose class antibiotics should only be prescribed by a clinician of higher rank. Restricted antibiotics can only be prescribed by the attending clinician or a doctor of higher rank. Unrestricted class antibiotics can be prescribed by any class of clinicians.

##### Leadership commitment

Antimicrobial management committee (AMC) was set up in all the 7 medical institutions, the members were mainly from the medical department, the hospital infection control department, or other related clinical departments such as the pharmacy department, the microbiology laboratory and the information department. Hospital associated Infection Management Committee was set up in hospitals except for community healthcare centers. The hospital leadership is in charge of the two committees.

##### Actions

The implementation of the administrative policy on antimicrobial management measures is in accordance with the documents issued by all seven medical institutions. Medical institutions with formal infection control department had infection management guideline.

In the area of microbiology, four tertiary hospitals are able to provide epidemiological reports on microbiology and susceptibility data.

All 7 medical institutions took administration-led measures to strengthen rational use of antibiotics, and 7 medical institutions took classified management of antibiotics and reviewed and gave feedback after prescription.

**Fig. 3:**
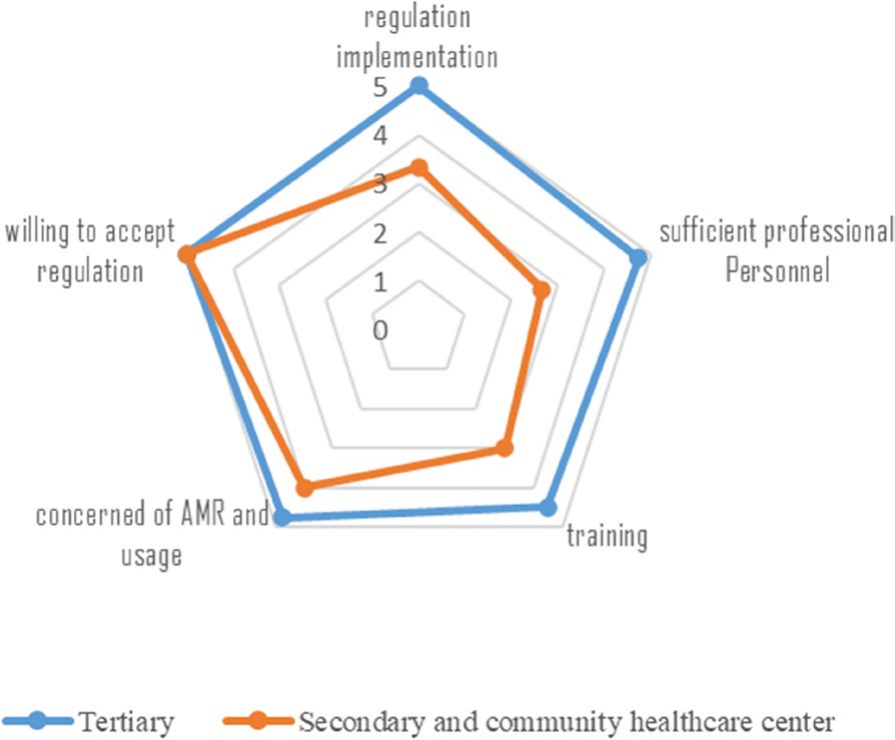
Attitudes and practice of doctors from tertiary, secondary and community healthcare center

The seven healthcare facilities including 4 tertiary hospitals, 1 secondary hospital and 2 community healthcare centers. The details of medical institution are in Table 1. According to the interview, attitudes and practices of clinicians from different level of medical institution differs in the following 6 dimensions (Fig. 3). Four clinicians from level III hospitals and two from level II and community health care centers work in medical institutions with strict or very strict management regulations, only 1 clinician from community health care center not sure about the implantation of regulations. Tertiary hospital clinicians thought they have sufficient professional personnel related to AMR containment, however, clinicians from small medical institution said they were more or less faced with the shortage of microbiologist and pharmacists. Big hospital clinicians said they have received intensive or frequent compulsory stewardship training, clinicians from community healthcare center received training less frequent by contrast. Tertiary hospital clinicians were more concerned of the status of AMR and usage of antimicrobial agents. All the interviewed clinicians were very willing to accept the National Action Plan related regulations such as antimicrobial stewardship.

**Table 1:** Details of medical institution

Characteristic	N
Level	
Tertiary hospital	4
Secondary hospital	1
Community healthcare centers	2
Type	
Specialized hospital	2
Integrated hospital	5
Location	
Provincial capital	6
others	2
Beds counts	
>2000	4
1000–2000	2
<100	2

##### Tracking

DDD value was the index with the highest frequency of use, followed by the number of days of antibiotic treatment, antibiotic resistance rate, medical expenditure and the implementation of management system.

Penalties for improper use of antibiotics include public disclosure of the list of departments and doctors, financial penalties, naming and shaming, suspension of prescribing authority and mandatory training of the doctors concerned.

### Themes

Factors impacting the NAP implementation in medical institutions following 6 key themes emerged from the interviews: Individual factors, leadership, multidisciplinary collaboration, patient factors, training and culture (Table 2).

**Table 2:** Description of the identified themes

Example of Quotes	Description	Subthemes	Themes
-	All clinicians, microbiologists, pharmacists, and nosocomial infection management personnel conveyed a robust willingness to endorse the National Action Plan. Across all professions, pharmacists scored highest for different dimensions of attitude. (Fig. 3)	Attitudes	Individual factors
-	Across all professions, pharmacists scored highest for self-learning. (Fig. 3)	Professional behavior	
*“As long as leaders pay attention, things can be done, whether it is antimicrobial stewardship or hospital associated infection control.” [nosocomial infection management personnel]*	A lack of awareness among managers of the importance and strategies for curbing antimicrobial resistance will hinder the successful adoption and implementation of national action plans in health facilities.	Administrative support	Leadership
*“To be honest, sometimes when we give advice to doctors, they don’t necessarily follow it.” [Pharmacist]*	Hospital managers recognized that recruiting specialist staff was not enough, as these staff needed to work together as a team, and many clinicians interviewed further emphasized that structural disintegration would hinder effective team work by antimicrobial management teams.	Multidisciplinary collaboration	Professional collaboration
*“Clinicians are more careful about the use of antimicrobial agents than surgeons, and I think we need more cooperation because clinicians need some guidance on how to interpret reports of sensitivity test.” [Microbiologist]*			
*“Antimicrobial drugs are a double-edged sword, irrational use will lead to drug resistance, But the reality of the situation you face is always complicated.” [Clinician]*	A significant concern for doctors revolves around the substantial pressure of responsibility.	Clinical experience	Patient factors
*“Patients sometimes ask me to prescribe antibiotics, but if I communicate with them, they are very compliant with my prescriptions” [Clinician]*	Respondents sometimes felt pressured to prescribe antibiotics, especially when they felt there was no need for patients or their relatives to use antibiotics.	Patient preferences	
*There always this kind of training, it is irregularly. It’s mainly clinical cases discussion. We are very busy. Our schedule is too full. [clinician]*	Training is mainly irregularly and unsystematic and comes mainly from clinical cases discussion.	Irregularly and unsystematic	Training
*When there is a will, there is a way. When patient is convinced that antimicrobial agents are working for them, they will find a way to get them, so it is important to teach correct knowledge. [clinician]*	When patient get cold or even other disease, the first reaction is to take antimicrobial agents.	Antimicrobial agents use culture	Culture

A total of 20 interview participants were involved in this interview, with an average age of 46. Out of these, 7 were clinicians, 3 were microbiologists, 3 were pharmacists, 3 were nosocomial infection management personnel, 4 were administrators. More than 70% of the interviewees had Mater, MD or PhD degree. 80% of the interviewees were working in the related field for more than 15 years (Table 3).

**Table 3:** Basic information of interviewees

Characteristics		N (%)
Age(Years): 46 ± 7 (mean ± S.D.)		
≥ 45		11 (55%)
35–44		6 (30%)
≤ 34		1 (5%)
Education		
Doctor		7 (35%)
Master		7 (35%)
Bachelor		6 (30%)
Years of working (years): 21 ± 8 (mean ± S.D.)		
≥15		16 (80%)
10–14		4 (20%)
<10		0 (0%)
Profession		
Clinician		7 (35%)
Microbiologist		3 (15%)
Pharmacist		3 (15%)
Nosocomial infection management personnel		3 (15%)
Administrator		4 (20%)

#### Individual factors

##### Attitudes towards implementation of the national action plan across professions

Across all professions, pharmacists scored highest for different dimensions of attitude. All clinicians, microbiologists, pharmacists, and nosocomial infection management personnel conveyed a robust willingness to endorse the National Action Plan. Among the interviewees, 80% expressed being “constantly concerned” about the current state of antimicrobial resistance (AMR) within their medical institutions. Notably, this heightened level of concern was observed in only 42.9% of clinicians. The utilization of antimicrobial agents emerged as a predominant concern for pharmacists, clinicians, and microbiologists. In contrast, nosocomial infection management personnel reported occasional rather than frequent concerns in this regard (Fig. 3).

##### Practice towards implementation of the national action plan across professions

**Fig. 4:**
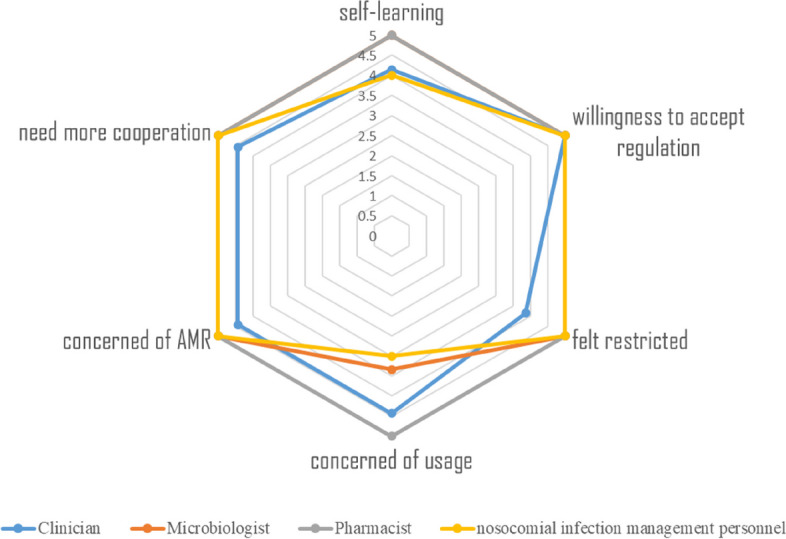
Attitudes and practice of clinicians, microbiologist, pharmacist and nosocomial infection management personnel

Across all professions, pharmacists scored highest for self-learning. Among all four specialties, only clinicians (score = 3.86) reported moderate restrictions on the prescribing of antimicrobials during the implementation of regulations governing antimicrobial agents (Fig. 4). All professions felt they need more cooperation with other professional. Clinicians were the only professionals who expressed relatively minor restrictions in their daily work, mainly with regard to the prescription of antimicrobial drugs, with moderate restrictions during the implementation of the regulations governing antimicrobial drugs. In terms of self-learning, 2 of 7 clinicians said they proactively acquired knowledge about AMR or antimicrobial agents only sometimes. 1 of 3 HAI management personnel acquired knowledge about AMR or antimicrobial agents by self-learning less frequency.

#### Leadership

A lack of awareness among managers of the importance and strategies for curbing antimicrobial resistance will hinder the successful adoption and implementation of national action plans in health facilities. In addition, the management team was not convinced of the benefits of antimicrobial management and infection control in terms of antimicrobial consumption, reduction of antimicrobial resistance, and improved patient outcomes. This is critical because a lack of support and commitment from top management has been identified as a significant barrier to taking and implementing action in hospitals. Top management here can improve the visibility of the hospital’s strategy and strengthen adherence to its policies. The following statements illustrate this view:

*“As long as leaders pay attention, things can be done, whether it is antimicrobial stewardship or hospital associated infection control.”* [nosocomial infection management personnel].

“*Thanks to leaders’ attention, we are doing better and better in antimicrobial stewardship.”*[pharmacist].

Given the limited capacity of managers to pay attention to a problem and allocate resources, priority setting within hospitals represents the importance of focusing on issues such as short-term needs and reducing immediate risks and the long-term impact of drug resistance.

*“With the advent of DRG payment methods, hospitals have entered the era of cost control, the extra cost of a patient’s infection will be covered by the hospital not to mention a drug-resistant infection, containment of resistance will be taken to a higher strategic level.”* [Administrator].

#### Multidisciplinary collaboration

The lack of relevant professionals was also identified as a major obstacle to action taken and implemented by medical institutions. In particular, the lack of clinical pharmacists was cited as a reason for the limited degree of action taken by hospitals. In particular, it says clinical pharmacists will be able to advise on the appropriate use of antibiotics and, most importantly, track policy enforcement and strengthen prescribing practices. The lack of microbiologists and laboratory equipment can also be an obstacle to the implementation of national action plans.

*“We only have one pharmacist; I don’t know if he can handle all this.”* [Non-tertiary hospital Clinician].

*“We only do routine blood tests. Our clinical laboratory can’t do blood culture test.”* [Non-tertiary hospital Clinician].

*“In my opinion, for hospitals, infection control measures are the key measures to prevent the nosocomial transmission of drug-resistant bacteria.”* [Clinician].

However, hospital managers recognized that recruiting specialist staff was not enough, as these staff needed to work together as a team, and many clinicians interviewed further emphasized that structural disintegration would hinder effective team work by antimicrobial management teams. In addition, there appears to be a need for pharmacists, microbiologists, and hospital-acquired infection epidemiologists to coordinate antimicrobial administration with clinicians.

“*We are like sentinels in the battle against drug resistance.”* [Microbiologist].

*“We welcome professional advice from clinical pharmacists on medication, but the work of pharmacists in clinical departments seems to have stopped recently.”* [Clinician].

*“Clinicians are more careful about the use of antimicrobial agents than surgeons, and I think we need more cooperation because clinicians need some guidance on how to interpret reports of sensitivity test.”* [Microbiologist].

*“In addition to hand hygiene, I think I really need guidance from the hospital infection department on cleaning and disinfecting our environment.”* [Clinician].

*“Doctors have complained to me about the effectiveness of prescribing drugs based on sensitivity test results.”* [nosocomial infection management personnel].

*“I think the working mechanism of clinical pharmacists should be further improved, such as remuneration.”* [Pharmacist].

Clinicians have certain clinical autonomy due to their specific professional knowledge, which leads to the inability of managers to overcome the authority of clinicians in the clinical field.

*“To be honest, sometimes when we give advice to doctors, they don’t necessarily follow it.*” [Pharmacist].

#### Patient factors

The majority of respondents are well-informed about and apprehensive regarding bacterial resistance, acknowledging that inappropriate antibiotic prescribing can contribute to this issue. However, a significant concern for doctors revolves around the substantial pressure of responsibility. Clinicians often hesitate to modify antibiotic prescriptions or shorten treatment durations in alignment with guidelines, fearing potential deterioration in patients’ conditions. In such instances, doctors may adopt a defensive approach in prescribing, driven by apprehensions of legal or administrative repercussions.

Another notable concern is that the risks and benefits of antibiotic prescribing are predominantly considered for current patients rather than extending this consideration to future ones. Influencing clinicians to modify their antimicrobial prescribing practices can be a challenging endeavor due to inadequate enforcement of guidelines, the weight of clinician responsibilities, or personal characteristics and behaviors.

*“Antimicrobial drugs are a double-edged sword, irrational use will lead to drug resistance, But the reality of the situation you face is always complicated.”* [Clinician].

Respondents sometimes felt pressured to prescribe antibiotics, especially when they felt there was no need for patients or their relatives to use antibiotics.

*“Patients sometimes ask me to prescribe antibiotics, but if I communicate with them, they are very compliant with my prescriptions”* [Clinician].

#### Training

Current education around antibiotics was described by respondents as problematic because of: limited time available to clinicians and an unwillingness to participate in the educational process due to learning fatigue. In addition, antibiotic prescribing covers a very wide range of specialties, so the messages that need to be conveyed to different specialties and professional groups are very broad and complex, which is considered to be a significant barrier to adequate antibiotic education.

*“There always this kind of training, it is irregularly. It’s mainly clinical cases discussion. We are very busy. Our schedule is too full.”* [Clinician].

#### Culture

The public does not know enough about antibiotics. Respondents reported feeling pressure from patients and parents to prescribe antimicrobials. They feel that sometimes there is no time to solve the problem, hindering the opportunity for patient education.

*“Sometimes patients who are used to certain antibiotics will ask me to prescribe them, sometimes patients require an intravenous drip, sometimes patients are adamant against antibiotics because they’ve heard they’re bad.”* [community health center clinician].

*“When there is a will, there is a way. When patient is convinced that antimicrobial agents are working for them, they will find a way to get them, so it is important to teach correct knowledge.”* [community health center clinician].

## Discussion

China’s Ministry of Health, in collaboration with 13 other ministries, has unveiled the National Drug Resistance Action Plan (NAP), serving as the official response to the World Health Organization’s global action plan and the initiatives undertaken by major global political groups for drug resistance control 2. The NAP outlines strategies for healthcare institutions to integrate antimicrobial management and infection control into their hospital management systems. The response measures implemented by the local health administration department in Hubei province regarding the NAP primarily involve the reinforcement of antibacterial drugs and the oversight of clinical application classification catalogue. Notable initiatives include the clinical use of antibiotics special rectification campaign (2015–2017), the establishment of the clinical pharmacy quality control center and expert committee, the formation of surveillance networks of Antibiotic Use and bacterial resistance, and the execution of health education programs.

This study is centered on exploring the perspectives of medical institutions, aiming to comprehend the external policy environment for NAP implementation, internal organization collaboration, and stakeholders’ perceptions and attitudes towards the NAP. Additionally, the investigation seeks to identify factors influencing the implementation of the NAP within medical institutions.

Medical institutions’ effort to combat antimicrobial resistance primarily focus on two key aspects: antimicrobials stewardship and infection prevention. A major strategy outlined in the NAP involves enhancing the construction of AMR control system, with a particular emphasis on reinforcing the management of hospital infections. Many antimicrobial organisms found in hospitals are a direct result of healthcare interventions, underscoring the critical importance of robust infection prevention and control guidelines. The NAP recognizes the synergy between infection prevention and antimicrobial stewardship. Coordinated and targeted prevention strategies play a crucial role in halting the spread of multi-drug-resistant organisms. Furthermore, measures for nosocomial infection prevention and control contribute to the reduction and optimization of antibiotic use. In the investigated tertiary hospitals, committees on the Rational Use of Antimicrobial Drugs and Hospital Infection Management, with hospital leaders serving as committee directors, have been established. These hospitals have also implemented multidisciplinary cooperation mechanisms, forming a working group comprising clinical departments, forming a working group comprising clinical department, clinical microbiology, pharmacy and hospital infection management. A study evaluating antibiotic management in China’s Class A tertiary hospitals underscores the need to strengthen the rational use of antibiotics through measures [7]. In a separate study, a multi-department collaborative management process for antibiotics in a hospital was introduced, involving the establishment of a collaborative management system by the information center based on hospital infection management department procedure. Clinical department directors and pharmacists sequentially approved the application proces the system [8]. Our study reports positive responses to antimicrobial control measures in a Chinese tertiary hospital. It elucidates that the antimicrobial program including administrative management, especially information management, was effective in reducing antibiotic consumption and lessening antibiotic resistance [9]. Our study echoes positive responses to antimicrobial control measures in a Chinese tertiary hospital. It highlights the effectiveness of the antimicrobial program, particularly in administrative and information management, in reducing antibiotic consumption and mitigating antibiotic resistance.

Our study, although regional in scope, stands as the pioneering effort to interpret the factors influencing the implementation of the National Drug Resistance Action Plan (NAP). We have identified six pivotal themes-individual factors, leadership, professional interactions, patient factors, training and culture-that have had a significantly influenced on the successful implementation of the NAP.

Our findings reveal a unanimous and robust willingness among participants, with 100% expressing their readiness to embrace the National Action Plan. This consistency aligns with observations from various studies, predominantly those focused on antimicrobial stewardship (AMS) worldwide. For instance, Feng et al.’s research noted a positive attitude among community pharmacy staff towards antimicrobial management programs [10]. Australian Emergency Department clinicians perceive injudicious antimicrobial use as problematic [11]. Moreover, there was a noticeable improvement in doctors’ willingness to adhere to regulations from 2012 to 2016 [12]. Pharmacists in government hospitals reported an outstanding adoption of Antimicrobial Stewardship (AMS) programs in a qualitative analysis [13].

Our study highlight leadership as a significant factor, aligning with findings from other studies. In Saudi Arabia, the implementation antimicrobial stewardship faces challenges, with the antimicrobial management team identifying barriers such as the lack of adoption of policies and guidelines from the Ministry of Health and hospital administration [14]. Health managers in the region recognize the fundamental importance of antimicrobial stewardship programs and understand the extensive benefits of implementing and institutionalizing such programs in hospitals [15]. To bolster credibility, it is essential to demonstrate the impact of the antimicrobial stewardship program on clinical outcomes and costs. Furthermore, engaging senior leaders is crucial. By obtaining their endorsement and investment in the antimicrobial stewardship program, we can enhance the credibility of antimicrobial stewards. This, in turn, augments their ability to influence the effective uptake of antimicrobials [16].

Professional interactions were the key factor which can facilitate the NAP implementation in our study. Other studies demonstrated that hindrances encompass team disintegration, poor communication, recruitment challenges, inadequate education and training, and a dearth of health information technology. A survey conducted in Norway emphasized the pivotal role of microbiological test results and newly published national antimicrobial guidelines, identified by infectious disease experts, as key factors influencing antimicrobial prescribing practices [17]. The practice of antimicrobial stewardship is not implemented and well developed as demonstrated by lack of core complementary health services, the importance of teamwork is emphasized [18]. Pharmacist-driven antimicrobial stewardship in an intensive care unit decreased patient mortality and the emergence of multidrug resistance, and optimized antimicrobial agent use [19]. Participants also identified a lack of clinical pharmacists and highly skilled staff as the main limitations in antimicrobial stewardship program implementation [13]. Hospital managers’ report considerable inter-professional challenges to actualizing antibiotic optimization and governance [20]. Pharmacists are core AMS team members where there is an ongoing need to align continuing education for health professionals with realities of practice [21]. Pharmacy-supported interventions were one of the quality indicators of hospital antimicrobial stewardship programme [22].

Our study also concluded that the concern of patient is a contributing factor. The accessibility of restricted antibiotics by clinicians, driven by concerns about patient outcomes, emerged as a potential challenge to effective regulation [12].Clinicians’ apprehensions about accountability further hinder the adoption of antimicrobial management [14]. Patient assessment, informal training by experienced colleagues, and infectious disease specialists replacing managers in promoting prudent prescribing policies, also influenced prescribing practices [17].

Continuing education for healthcare professionals should consistently align with the practical aspects of their work [21]. The development of antimicrobial stewardship programs in Norway should include systematic training on prudence as a crucial factor to be addressed [17]. While recollection of formalized undergraduate teaching and hospital guidelines have a minimal influence, participants express the need for undergraduate teaching to be more practical and presented in a way that is easily applicable to on-ward situations [23].

This study has limitation that should be taken into consideration when interpreting the results. The respondents answered our questions through recall, and there is a possibility of recall bias.

## Conclusions

Based on our analysis, practitioners from medical institutions have shown strong support for the National Antibiotic Plan (NAP). Health administration efforts has in implementing the NAP have yielded positive results, with a notable decline in the prescription rate antibiotics in medical institutions from 2016 to 2019 in Hubei. Participants demonstrated awareness and concern about antimicrobial resistance, expressing high support for the NAP. Tertiary hospitals have successfully established multidisciplinary cooperation mechanisms. Six main themes identified as both barriers and facilitators to the implementation of the NAP in the medical institutions: individual factors, leadership, multidisciplinary collaboration, patient factors, training and culture. Considering of practitioners’ perceived barriers and facilitators is crucial for enhancing implementation of the NAP to contain antimicrobial resistance. Future research should include relevant quantitative studies to further investigate and validate these findings.

### Supplementary Information

**Additional file 1.**

## Data Availability

The original contributions presented in the study are included in the article, further inquiries can be directed to the corresponding author/s.
