# Supplementary material for: A qualitative study of the factors impacting implementation of the national action plan to contain antimicrobial resistance (2016–2020) in medical institutions
Source: BMC Health Serv Res. 2024 Jan 22;24:120. doi: 10.1186/s12913-023-10404-y (PMC10804545; doi:10.1186/s12913-023-10404-y)
Supplement: Supplementary file 1 — Additional file 1. [file 12913_2023_10404_MOESM1_ESM.docx]

**A** **qualitative study of the factors impacting implementation of** **the National Action Plan to Contain Antimicrobial Resistance (2016–2020) in medical institutions**

**Yun Tao^a,1^, Ying Wang^b,1^, Yu Zhang^a^, Yong Han^a^, Jiajia Feng^a^, Hong Cheng^c,^ *, Likai Lin^a,^ ***

^a^ Hospital Management Institute of Wuhan University, Zhongnan Hospital of Wuhan University, Wuhan, Hubei, 430071, China.

^b^ Department of Infectious Diseases, Zhongnan Hospital of Wuhan University, Wuhan, 430071, Hubei, China.

^c^ Department of Pharmacy, Zhongnan Hospital of Wuhan University, Wuhan, 430071, Hubei Province, China.

**^*^** Corresponding author address: Zhongnan Hospital of Wuhan University, Wuhan, 430071, China.

E-mail addresses: linlikai_1963@163.com, chenghong@znhospital.cn

^1^ These authors contributed equally to this work.

**Supplementary material**

**Semi-structured interview guides**

|  | **Personnel of health management department of medical institution** |
| --- | --- |
| 1 | Could you give us a brief self-introduction? Let us know your basic information (the interviewee's basic information, including name, age, education level, years of work, current position, etc.) |
| 2 | What is the current organizational structure of the pharmaceutical administration department of the health administration department? Do higher authorities have guidelines on antibiotic abuse? Is there a specific body that regulates the use of antibiotic drugs in health care Settings? |
| 3 | Are there guidelines for medical institutions on the standardized use of antibiotics? What are the main ways to supervise the rational use of antibiotics in medical institutions? |
| 4 | In what ways can the general public obtain antibiotic drugs? What do you think of the public's awareness of antibiotics? |
| 5 | What activities do health authorities carry out on a daily basis to raise public awareness about antibiotics? |
| 6 | Do you think antibiotics are overused? (What causes overuse?) What are the obstacles in the fight against antibiotic resistance? How do you think it can be improved? |

|  | **Clinician** |
| --- | --- |
| 1 | Could you give us a brief self-introduction? Let us know your basic information (the interviewee's basic information, including name, age, education level, years of work, current position, etc.) |
| 2 | What antibiotics have you used the most in the past six months? What are your main reasons for prescribing such antibiotics? |
| 3 | Will the pharmacy department of the hospital or the local health commission restrict the use of antibiotic drugs? How to limit？ |
| 4 | Does your medical institution have any guidelines for the standard use of antibiotics? Have you had any medication training in the past year? How many training sessions? |
| 5 | During the visit, have any patients asked you to prescribe antibiotics? How do you respond if a patient insists on an antibiotic prescription? If prescribed, will you give a detailed explanation of the medication? |
| 6 | What are the main considerations for prescribing antibiotics to patients? Consider evidence of bacterial infection, not viral infection. Blood routine, chest film (required, outpatient may not perform). Doctor inertia thinking (grassroots with experience medicine). |
| 7 | What do you think of the public's awareness of antibiotics? Do you inform patients about the use of antibiotics during the visit? |
| 8 | Do you think the current use of antibiotics is reasonable? (What causes overuse?) What are the obstacles in the fight against antibiotic resistance? How do you think it can be improved? |

|  | **Personnel in hospital Infection department or pharmacy department** |
| --- | --- |
| 1 | Could you give us a brief self-introduction? Let us know your basic information (the interviewee's basic information, including name, age, education level, work unit, current position, etc.) |
| 2 | Does the superior competent health department formulate corresponding guidelines for the standardized use of antibiotics in hospitals? Implement it? |
| 3 | What is the current organizational structure of the pharmaceutical administration department in the hospital? Is there a special department that regulates the use of antibiotic drugs by clinicians? Does the hospital have a monitoring/evaluation system for antibiotic resistance? |
| 4 | What kinds of antibiotics are commonly used in hospitals? why |
| 5 | Under what circumstances can a clinician prescribe antibiotics to a general patient, and are there standards in place? |
| 6 | Does the hospital train health care staff on antibiotic use? How often is the training? |
| 7 | Do you think the current use of antibiotics is reasonable? (What causes overuse?) What are the obstacles in the prevention and control of antibiotic resistance in hospitals? How do you think it can be improved? |
| 8 | What do you think is the current status of antibiotic resistance? What do you think are the reasons for this phenomenon? |

|  | **Hospital administrator** |
| --- | --- |
| 1 | Could you give us a brief self-introduction? Let us know your basic information (the interviewee's basic information, including name, age, education level, work unit, current position, etc.) |
| 2 | Is there a special department in the hospital that monitors the use of antibiotics? Does the hospital have a monitoring/evaluation system for antibiotic resistance? What does the system monitor (what information is collected)? |
| 3 | Does the superior competent health department formulate corresponding guidelines for the standardized use of antibiotics in hospitals? (Asking for details) How is the implementation going? |
| 4 | What kinds of antibiotics are commonly used in hospitals? Why are these drugs often used? |
| 5 | Under what circumstances can a clinician prescribe antibiotics to a general patient, and are there standards in place? How is the implementation going? Is there a limit to the amount of antibiotics used in the department or clinic? (e.g., performance-related) |
| 6 | What do you think is the level of awareness of antibiotics among clinicians? |
| 7 | Does the hospital train health care staff on antibiotic use? How often is the training? Content and form of training |
| 8 | Do you think the current use of antibiotics is reasonable? (What causes overuse?) What are the obstacles in the prevention and control of antibiotic resistance in hospitals? How do you think it can be improved? |
| 9 | What advice do you have for strengthening antibiotic management and curbing antibiotic resistance? |

**Structured interview guides**

|  | **Attitudes and practice attitudes and practice (personal)** |
| --- | --- |
| 1 | Willingness to accept regulation (Score 1-5, The higher the score, the more agreement) |
| 2 | Felt restricted (Score 1-5, The higher the score, the more agreement) |
| 3 | Need more cooperation (Score 1-5, The higher the score, the more agreement) |
| 4 | Concerned of AMR (Score 1-5, The higher the score, the more agreement) |
| 5 | Concerned of antibiotic use (Score 1-5, The higher the score, the more agreement) |
| 6 | Self-learning (Score 1-5, The higher the score, the higher the frequency) |
|  | **Attitudes and practice attitudes and practice (medical institution)** |
| 1 | Regulation implementation (Score 1-5, The higher the score, the more agreement) |
| 2 | Sufficient professional personnel (Score 1-5, The higher the score, the more agreement) |
| 3 | Training (Score 1-5, The higher the score, the higher the frequency) |
| 4 | Concerned of AMR and usage (Score 1-5, The higher the score, the more agreement) |
| 5 | Willing to accept regulation (Score 1-5, The higher the score, the more agreement) |
